# Beyond translation: A review into preserving human connection in AI-mediated health language interpretation

**DOI:** 10.1371/journal.pdig.0001295

**Published:** 2026-03-12

**Authors:** Kristina M. Kokorelias, Kateryna Metersky, Christina Reppas-Rindlisbacher, Areej Al-Hamad

**Affiliations:** 1 Section of Geriatric Medicine, Department of Medicine, Sinai Health System and University Health Network, Toronto, Ontario, Canada; 2 Department of Occupational Science and Occupational Therapy, Temerty Faculty of Medicine, University of Toronto, Toronto, Ontario, Canada; 3 Rehabilitation Sciences Institute, Temerty Faculty of Medicine, University of Toronto, Toronto, Ontario, Canada; 4 KITE- Toronto Rehabilitation Sciences Institute, Toronto, Ontario, Canada; 5 Daphne Cockwell School of Nursing, Toronto Metropolitan University, Toronto, Ontario, Canada; 6 Institute for Health Policy, Management, and Evaluation, University of Toronto, Toronto, Ontario Canada; Liverpool John Moores University - City Campus: Liverpool John Moores University, UNITED KINGDOM OF GREAT BRITAIN AND NORTHERN IRELAND

Patients who do not speak the national language(s) of a country where they are seeking care face serious risks to their health from language barriers. The risks include misdiagnoses, inappropriate treatment plans, more frequent hospital readmissions, higher rates of infection, and in some cases preventable mortality [1]. These outcomes often stem from communication errors that result in unclear instructions, incomplete histories, and inadequate understanding, which together create serious concerns for both safety and quality of care [1]. Professional medical interpretation, usually offered in-person, by phone, or by video conferencing is known to improve care quality and patient outcomes [2]. However, interpreter services remain inconsistently available due to cost, scheduling delays, and workforce shortages.

At the same time, artificial intelligence (AI) translation tools such as Google Translate, DeepL, Microsoft Translator, and emerging clinical applications are becoming widely accessible in healthcare settings [3,4]. Their growing use raises an important question: how can health systems benefit from AI-mediated interpretation without compromising relational communication, trust, and equity?

In this paper, we use the term AI-mediated interpretation to refer to the use of artificial intelligence tools for either written translation of clinical materials (e.g., discharge instructions) or real-time spoken interpretation between clinicians and patients. AI-mediated interpretation is intended to supplement, not replace, professional human interpreters. We emphasize that most evidence currently pertains to written translation, and conclusions regarding real-time interpretation are limited by the sparse evidence base. We argue that AI-mediated interpretation should be used as a supplement to professional interpretation, not a replacement. Health systems must establish accuracy standards, clinical guidance, and equity safeguards before integrating these tools into routine care. This paper focuses specifically on clinician–patient communication, including history-taking, explaining diagnoses, discharge instructions, and emotionally sensitive conversations.

## AI translation performance in healthcare communication

A primary study evaluating sentence-level accuracy of AI and professional translations of English discharge instructions noted that *any* language assistance boosts comprehension, adherence and satisfaction versus ad hoc efforts [5]. Similarly, patients often report being reassured by even imperfect AI-mediated interpretation, whereas clinicians worry about the risks of errors and loss of nuance [6]. However, human interpreters are often scarce, delayed, or costly, leading clinicians to experiment with AI-based tools (e.g., Google Translate, DeepL, Microsoft Translator or specialized apps) for real-time interpretation [3,4]. Recent evidence shows these AI tools can produce highly accurate translated text in many cases, especially for common language pairs and simple text [3]. For example, Kong et al. (2025) evaluated ChatGPT-4 and Google Translate on English discharge instructions for Spanish and Chinese [7]Sentence-level accuracy exceeded 90%, meaning that over 90% of sentences were translated without errors that could affect patient understanding or clinical instructions [7]. However, AI performance dropped substantially for other languages, such as Russian where the GPT-4 fell to ~89% and Google to ~80% accuracy (with up to 6% of instruction sets containing potentially harmful errors) [7]. Lower accuracy for English-to-Russian translations likely reflects not language prevalence but the complexity of clinician-written sentences, including low readability, multi-clause structures, and atypical word usage, that can challenge current AI models. [7] Similarly, a study of pediatric discharge instructions found AI-based translation was non-inferior to human translation for Spanish but showed more errors and lower fluency for the languages of Chinese, Vietnamese and Somali [5]. By contrast, professional translators outscored AI on fluency in every language, and clinically impactful errors were far more common in AI translations outside of Spanish [5]. These findings are important for both AI design and clinical care, since AI systems are typically trained on widely used languages and may not perform as well for languages spoken by smaller, refugee, migrant, or Indigenous communities [8,9]. From a clinical standpoint, if health systems adopt AI translation as a default solution without accounting for this variation, the risk of communication error may shift disproportionately onto patients who already experience barriers to care. This raises both safety and equity concerns.

## Limits of AI-mediated interpretation in relational care

AI translation performs best with short, structured written content. Real-time spoken interpretation presents greater challenges. Clinical encounters require rapid two-way communication, interpretation of emotional tone, and sensitivity to cultural meaning. Current AI systems often miss idiomatic language, emotional nuance, and contextual cues. For example, in a systematic review of AI in clinical translation, Genovese et al. report that AI “often provides sufficiently accurate translations for brief, simple communications when translating from English,” but error rates climb for longer discussions, non-European languages, or reverse translation into English [6]. Similarly, Google Translate was found to frequently mistranslate critical clinical terms and produced unnatural phrasing that could mislead patients [10]. Similarly, early studies on voice-based interpreting tools show that machines often miss idiomatic expressions, emotional tone, and cultural nuance, that limits how well they can support natural communication [11]. These limitations are most concerning in situations where both accuracy and compassion are essential, such as conversations about mental health, reproductive health, sexually transmitted infections, cancer, dementia, or end-of-life care. If AI translation misses emotional tone or changes meaning, patients and families may not fully understand their condition or may feel misunderstood. Similarly, as translation may result in a lack of clinicians conveying empathy, patients and their families may feel unsupported at moments when trust and compassion are essential [12]. On the other hand, back-translation of AI-mediated translations into English retained only 36–76% of the original meaning for certain non-European languages, with some errors potentially altering the clinical intent of the instructions [6]. Therefore, while AI translations offer practical advantages, they should complement, not replace, human interpreters, especially when empathy and trust are critical. Clinicians may misinterpret the urgency or seriousness of patient or family concerns. For migrant patients navigating health systems while managing trauma histories, uncertain legal status, and limited familiarity with local norms, missing emotional cues or cultural nuance can increase vulnerability. This may discourage follow-up, delay treatment, or push families to rely on children or community members for interpretation, raising additional ethical concerns [13]. Technology that improves access to language assistance must not weaken relational trust.

## Written translation versus real-time interpretation

Most existing studies evaluate AI translation of written materials rather than real-time spoken interpretation. This distinction is important. Written discharge instructions can be translated, reviewed, and corrected. Clinical conversations require immediate interpretation and interpersonal sensitivity.

AI tools may therefore be appropriate for low-risk written communication but less reliable for complex clinical encounters. Professional human interpreters remain essential for complex, emotionally charged, or high-stakes clinical communication. Evidence supporting safe use in real-time interpretation remains limited, and caution is warranted.

## Clinical responsibility and implementation considerations

Even when AI-mediated translations are linguistically accurate, they may remain unsafe if patients cannot understand the content. Health literacy, how well patients can access, process, and use health information, must be considered alongside translation accuracy. Patient-facing materials should follow plain-language principles, using clear, jargon-free wording, short sentences, and logical formatting. Clinicians should employ teach-back methods or other verification strategies to confirm patient understanding, particularly for numerical risk, medication schedules, or complex instructions. Importantly, professional interpreters should be engaged whenever comprehension or patient safety is uncertain, regardless of AI tool performance. Integrating these safeguards ensures that AI translation supports not only linguistic accuracy but also meaningful understanding, helping preserve trust, safety, and human connection in clinical encounters.

Clinical guidance around the use of AI in interpretation is rapidly evolving [6]. Responsibility for translation errors remains unclear. Miscommunication could involve clinicians, healthcare organizations, technology developers, or regulatory systems. Without verification standards or clinical protocols, reliance on AI translation risks undermining communication quality and patient trust. Clear guidance is needed on when AI translation is appropriate, how accuracy should be validated, and how professional interpreters should remain integrated into care. The use of widely available consumer AI translation tools in clinical settings also raises important privacy and governance concerns. Clinicians and health systems should ensure that any AI-mediated interpretation tools used in patient care comply with organizational policies and legal requirements for PHI protection. Institutionally approved workflows, including secure platforms and documentation procedures, are essential to safeguard patient confidentiality while integrating AI tools into practice.

We propose three principles for responsible implementation:

- AI interpretation should be used primarily for low risk written communication that has been locally validated for accuracy.
- Professional interpreters should remain the standard for complex, sensitive, or high-stakes clinical encounters.
- Health systems should monitor language-related safety outcomes to ensure AI adoption does not widen inequities.

To make these recommendations immediately actionable, we propose a simple practice framework that stratifies clinical content by risk and outlines minimum safeguards for AI-mediated interpretation in patient-facing communication (Table 1).

**Table 1:** Practice framework: Using AI-mediated interpretation in clinician–patient communication.

Risk Level	Example Clinical Content	Recommended Use of AI	Minimum Safeguards
**Low-Risk**	Administrative notices, scheduling, general information	AI translation may be used with basic verification	- Verify language selection - Ensure content is understandable
**Moderate-Risk**	Routine discharge instructions, standard patient education materials	AI translation can be used **with verification** by clinician or staff	- Teach-back to confirm understanding - Document tool and language used - Use bilingual verification if feasible
**High-Risk**	Consent discussions, complex diagnoses, mental health, end-of-life, emotionally sensitive conversations	**Professional human interpreters required**; AI may be used only to support documentation or review	- Teach-back to confirm comprehension - Document AI use and any verification steps - Clear stop-rule: defer to interpreter if comprehension is uncertain - Maintain relational engagement and empathy

## Conclusion

AI-mediated translation has the potential to improve access to language support in healthcare, particularly for low-risk written materials such as discharge instructions. However, communication in clinical care is not only about linguistic accuracy, but it also requires empathy, trust, and cultural understanding.

AI tools can assist clinicians but cannot replace the relational role of professional interpreters in real-time, patient-facing encounters. Responsible integration will require clear accuracy standards, clinical guidance, and attention to equity. Preserving human connection must remain central as digital interpretation technologies evolve, and caution is warranted when applying AI to complex, emotionally sensitive, or high-stakes communication.
